# Using theories of sexual selection and sexual conflict to improve our understanding of plant ecology and evolution

**DOI:** 10.1093/aobpla/plv008

**Published:** 2015-01-22

**Authors:** Åsa Lankinen, Kristina Karlsson Green

**Affiliations:** Swedish University of Agricultural Sciences, Plant Protection Biology, PO Box 102, S-230 53 Alnarp, Sweden

**Keywords:** Mating-system evolution, plant immunity, pollen competition, pollen–pistil interaction, sexual conflict, sexual selection

## Abstract

Today it is accepted that the theories of sexual selection and sexual conflict are general and can be applied to both animals and plants. However, potentially due to a controversial history, plant studies investigating sexual selection and sexual conflict are relatively rare. Moreover, these theories and concepts are seldom implemented in research fields investigating related aspects of plant ecology and evolution. Even though these theories are complex, and can be difficult to study, we suggest that several fields in plant biology would benefit from incorporating and testing the impact of selection pressures generated by sexual selection and sexual conflict. Here we give examples of three fields where we believe such incorporation would be particularly fruitful, including (i) mechanisms of pollen–pistil interactions, (ii) mating-system evolution in hermaphrodites and (iii) plant immune responses to pests and pathogens.

## Abstract (precis)

Sexual selection and sexual conflict theories that explain evolution of sexual behaviour are often an integral part of animal studies investigating diverse research questions, e.g. in behaviour, ecology, physiology and immunology. In plants, however, relatively few studies investigate sexual selection and sexual conflict. In this review we discuss how taking these theories into account can be useful not only for our understanding of plant reproductive strategies but also in related research areas, including i) mechanisms of pollen-pistil interactions, ii) mating-system evolution in hermaphrodites and iii) plant immune responses to pests and pathogens.

## Introduction

Theories of ecology and evolution are often based on general principles and can be applied across taxa. However, due to research tradition we can find that some research questions are considered more important in some taxa than in others. One such example is theories of sexual selection and sexual conflict, which is a large and active field in animals while treated as relatively insignificant in plants.

Application of sexual selection theory to plants in the late 1970s and early 1980s (Charnov 1979; Willson 1979; Queller 1983) generated a wealth of research and also criticism (reviewed in Skogsmyr and Lankinen 2002; Moore and Pannell 2011). Most concern was based on a mismatch between how to test for sexual selection and the function of plant growth and reproduction (Lyons *et al.* 1989), and also a general unwillingness to fit plants into a concept developed for animals (Grant 1995) and unclearness about how to define sexual selection in plants (see Skogsmyr and Lankinen 2002; Moore and Pannell 2011). In fact, this controversy resulted in that the term ‘sexual selection’ was avoided in research articles that investigated sexual selection in plants (Willson 1994). Today, the criticism to sexual selection in plants is no longer apparent in the literature, which may reflect that this theory is accepted among botanists. However, relatively few current studies do test this theory in plants compared with in animals. We estimated the difference between animal and plant studies by performing a survey in Web of Science, based on articles published in the last 10 years (i.e. between 2004 and 2014, date of search: 8 December 2014). The search term ‘sexual selection plant*’ obtained only 1731 articles while the search term ‘sexual selection animal*’ generated 13 763 hits, i.e. an almost eight times higher number of articles on sexual selection in animals. Note that this is a very rough estimation where we have not manually gone through all hits to exclude irrelevant articles; e.g. when including the term ‘plant’ we also obtain articles where it is actually animals that are the focus but these animals feed or live on a plant. Thus there might be errors in numbers for both the animal and the plant side of the searches and most likely we have overestimated the number of articles on sexual selection in plants. The presumably more precise comparable terms ‘sexual selection pollen’ (or ‘sexual selection pollen competition’) and ‘sexual selection sperm’ (‘sexual selection sperm competition’) generated 284 (67) and 2246 (1506) hits, respectively, which gives an estimate of about 8 (22) times more studies on the animal side.

It is also noteworthy that development of sexual selection theory into the theory of sexual conflict (Parker 1979; Chapman *et al.* 2003), which is widely investigated in insects, was only considered relatively recently in plants (Bernasconi *et al.* 2004; Lankinen *et al.* 2006; Prasad and Bedhomme 2006; Delph and Herlihy 2012). In Web of Science, the search term ‘sexual conflict plant*’ and ‘sexual conflict animal*’ finds 280 and 3311 articles, respectively, during the last 10 years (2004–14) thus around 12 times more studies made on animals (please note that this was an equally rough survey as above). The terms ‘sexual conflict pollen’ (‘sexual conflict pollen competition’) generates 46 (13) hits, while ‘sexual conflict sperm’ (‘sexual conflict sperm competition’) gives 742 (489) studies, which indicates that these areas of research are about 16 (38) times more common in animal biology.

Moreover, the theories of sexual selection and sexual conflict do not appear to be integrated in studies with the aim to learn about plant ecology and evolution in related research areas, i.e. to improve our understanding of plant fitness in general. This is in contrast to studies in animals, where these theories often are integrated in other fields, such as ecology, behaviour, physiology and immunology (e.g. Eldakar *et al.* 2009; Green *et al.* 2014; Martínez-Padilla *et al.* 2014; van Lieshout *et al.* 2014).

Here, our aim is to discuss how taking the theories of sexual selection and sexual conflict into account have the potential to improve our general understanding of plant ecology and evolution. We will give a short introduction to sexual selection and sexual conflict in plants. We will then point out three research fields in which we think it may be particularly useful to consider these theories, including (i) mechanisms of pollen–pistil interactions, (ii) mating-system evolution in hermaphrodites and (iii) plant immune responses to pests and pathogens. Rather than providing a complete review of the literature we will use specific examples and mainly focus on pollen competition in the pistil, including results from one of the author's own studies on *Collinsia heterophylla* (Plantaginaceae).

## Sexual Selection and Sexual Conflict in Plants

### Sexual selection

**Figure 1.**
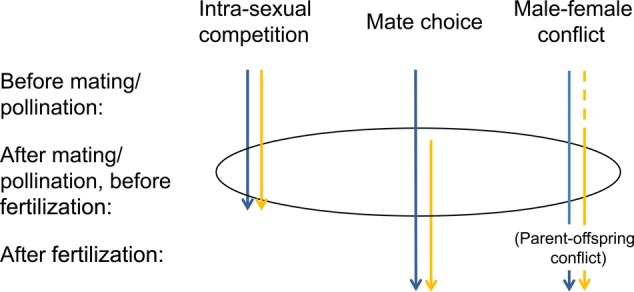
A schematic description of when during the life cycle to expect intra-sexual competition, mate choice and male–female conflict in animals (blue arrows) and plants (yellow arrows). Life cycle stages are represented by (i) before mating or pollination, (ii) after mating/pollination but before fertilization, i.e. the prezygotic stage, (iii) after fertilization. Sexual conflict after fertilization can be viewed as parent–offspring conflict (Queller 1994). In contrast to in animals, in plants the opportunity for mate choice before mating/pollination is less. However, intra-sexual competition can occur at this stage (e.g. involving traits that attract pollinators) and there is some possibilities for sexual conflict [mainly intra-locus (= dashed line), e.g. over traits that favour pollination success]. In plants most opportunities for sexual selection and sexual conflict occur at the prezygotic stage. In animals, post-copulatory mate choice is referred to as ‘cryptic female choice’ defined as ‘non-random paternity biases resulting from female morphology, physiology or behaviour that occur after coupling’ (Pitnick and Brown 2000), thus including both active and passive processes. Cryptic female choice would be applicable to post-pollination female choice in plants. This term, however, is not commonly used in plants, presumably because there has been no need to separate the choice mechanism after pollination from that before pollination.

Sexual selection is the idea of how traits can be favoured by selection because they increase reproductive success in competition with conspecific individuals of the same sex, either through intra- or inter-sexual (mate choice) competition (Darwin 1871) (Box 1). Sexual selection in animals—both in animals with separate sexes and hermaphrodites—is believed to be most important before mating, but can also take place after mating but before fertilization (during sperm competition), and after fertilization (Andersson 1994; Leonard 2006) (Fig. 1). In plants, where hermaphroditism is the most common breeding system, sexual selection can occur before pollination, when traits favouring pollinator attraction or optimal attachment on pollinators can increase pollen transfer to conspecific stigmas (Delph and Ashman 2006; Biernaskie and Elle 2007; Cocucci *et al.* 2014). Even though mating is done by proxy, involving a vector for pollen transfer, there is opportunity for both sexes or sexual functions to enhance their reproductive success (e.g. pollen export and import) relative to conspecifics (cf. interaction-independent sexual selection, Murphy 1998). Sexual selection in plants can also take place after fertilization, when sex-limited expression of traits influence allocation to developing fertilized ovules (Queller 1984). However, the potential for intra- or inter-sexual selection in plants is the greatest during pollen competition in the pistil, which is the equivalent to sperm competition and post-copulatory sexual selection in animals (Fig. 1). At this stage of the life cycle, pollen grains from different male parents are in contact with each other and with the female tissue, allowing both male–male competition and female choice by screening of pollen in the pistil. Also, hermaphrodite plants show a division between the sexual functions, pollen, i.e. the male gametophyte vs. the female gametophyte within the ovule. Here we can expect pollen traits that enhance competitive ability, such as pollen tube growth rate (Snow and Spira 1991; Pasonen *et al.* 1999), pollen germination rate (Austerlitz *et al.* 2012) or an ability to hinder other pollen, e.g. by chemical interference (Marshall *et al.* 1996; Varis *et al.* 2010). Pollen traits can also be influenced by pistil traits that enhance pollen competition providing an ability to sort among pollen, e.g. a long style (Mulcahy 1983; Ramesha *et al.* 2011), a large stigmatic surface (Armbruster 1996; Rodrigo *et al.* 2009) or delayed stigma receptivity and fertilization (Galen *et al.* 1986; Dahl and Fredrikson 1996). Note that pistil ability to favour some pollen over others does not require a cognitive ability. A recent study in *Arabidopsis* showed that female-determined mate choice was defined by four quantitative trait loci (QTLs) (Fitz Gerald *et al.* 2014), suggesting that the ability to sort among pollen can be genetically determined.

Box 1. **History and definition of sexual selection.**

Darwin first suggested the theory of sexual selection in order to solve a problem regarding his theory of natural selection (Darwin 1871); he found it difficult to explain existence of traits that reduced survival of its bearer. Because he observed that these traits were of importance during mating in animals, he proposed the idea of secondary sexual traits selected for in competition over mates as opposed to primary sexual traits, i.e. traits with a primary function in reproduction. The central idea of sexual selection was thus that mates represented a limited resource, favouring traits that increased the ability to access this resource before it was depleted. Sexual selection was therefore defined as reproductive success in comparison with individuals of the same sex. Darwin suggested two mechanisms of sexual selection—intra-sexual competition and mate choice. The latter was controversial because in the 19th century it was believed that you needed an education to be able to see and understand beauty, restricting such activities to ‘the most refined and civilized human beings’ (Zuk 2003; Hosken and House 2011). For this reason it was hard for scientists to believe that animals could make important choices regarding their mates. Even though this concern is not valid today, evolution of mate choice is still not completely understood (Jones and Ratterman 2009; Hosken and House 2011). Darwin's definition of sexual selection is very similar to the more modern definition of pre-copulatory sexual selection in animals (Andersson 1994; Jones and Ratterman 2009). However, along with the application of this theory to more taxa (hermaphrodite species, plants) and post-copulatory stages of the life cycle, the definition has been extended to include sexual reproductive functions (rather than sexes) and competition over fertilizations and embryo investment (Arnold 1994; Morgan 1994; Eberhard 1996; Skogsmyr and Lankinen 2002; Jones and Ratterman 2009; Moore and Pannell 2011). It should be noted that even though competition over fertilization at post-copulatory stages will involve competition between haploid sperm or pollen, the definition implies that sperm and pollen traits can only be sexually selected if these traits enhance reproductive success of the sperm- or pollen-producing diploid individual (Parker 1970; Willson 1979). Moreover, in hermaphrodites the same trait (e.g. flower size) can be sexually selected in both sexual reproductive functions at the same time provided that this trait provides a fitness advantage in competition with the same sexual function of other plant individuals (cf. Delph and Ashman 2006).

Already Darwin proposed that many traits can be influenced by both natural (viability or fecundity) and sexual selection, but stressed that distinguishing between them can be difficult. More modern definition of the relation between natural and sexual selection also suggest that traits can be influenced by either selective force or by a combination of both, and that discriminating between these forces has a value for our conceptual understanding and for separating (as a group) from neutral processes, i.e. genetic drift (Andersson 1994; Hosken and House 2011). For example, it is often particularly difficult to separate natural selection on pollen (with a primary function to fertilize the ovules) from that of sexual selection (the ability to fertilize the ovules ahead of pollen from other mates) (Moore and Pannell 2011). Post-mating mechanisms involving female ability to choose high-quality mates could be considered fecundity selection as it will not necessarily hinder other females from choosing these males (but note that such choice will lead to sexual selection on mates). In plants, it has been argued against the use of sexual selection instead of fecundity selection (Arnold 1994), while others have discussed that it is a matter of taste if fecundity or sexual selection should be used (Charlesworth *et al*. 1987). More recent suggestions include that competition over mates could refer to competition over both quantity and quality of mates (Skogsmyr and Lankinen 2002), and that any female effort beyond what is needed to ensure optimal fertilization in relation to available resources could be interpreted as sexual selection (Moore and Pannell 2011). In the current review we will use the more recent and broader definition of sexual selection.

Pistil traits that enhance pollen competition could evolve as a female choice mechanism (similar to cryptic female choice during sperm competition, Pitnick and Brown 2000). For example, enhanced pollen competition can be beneficial for the female function by favouring (i) fathers that will give rise to offspring with more competitive pollen (the Fisherian model, Lande 1981) or (ii) genetically superior fathers (good genes, the indicator model, Zahavi 1975; Hamilton and Zuk 1982). Pistil traits enhancing pollen competition could also evolve for a number of other reasons that are unrelated to sexual selection, for example favouring (iii) genetically more compatible fathers (Paschke *et al.* 2002) or (iv) increased diversity of offspring (Bernasconi *et al.* 2003). Note that relative choice, i.e. preference differs among females due to difference in compatibility of fathers, is discussed within the framework of sexual selection in animals (Lande 1981; Tregenza and Wedell 2000) while it is not in plants (Marshall 1998). Several suggested advantages of enhanced pollen competition furthermore involve the capacity to sort among haploid pollen (rather than among diploid fathers). Already in the 1940s it was observed that pollen selection can influence variability in the resulting sporophyte generation (Ter-Avanesian 1949). In the 1970s, Mulcahy (1971, 1979) proposed that (v) enhanced pollen competition was suggested to increase offspring quality due to selection of genetically superior pollen as expression of genes in pollen is overlapping with sporophytic genes (Hormaza and Herrero 1994; Borg *et al.* 2009). Even though based on haploid pollen, this mechanism shows some similarities to the ‘good genes’ mechanism (ii), where models have shown that female choice is most likely to evolve when the male preference trait reflects general condition of the father (Andersson 1994). Benefits of sorting among pollen also include pistil ability to avoid fertilization (vi) by self-pollen (cryptic incompatibility, Cruzan and Barrett 1993) or (vii) by poor quality self-pollen (Armbruster and Rogers 2004), reducing inbreeding depression (Lankinen and Armbruster 2007). Only sorting mechanisms leading to selection of pollen traits of diploid fathers are usually considered to be within the scope of sexual selection (see Box 1).

While there is empirical support for sexual selection of traits resulting from competition between the same sex or sexual function (usually males or pollen donors) both before and after pollination in several species, evidence for female choice, e.g. indirect sexual selection of pollen traits during pollen competition is scare and only tested in a few studies (see reviews by Skogsmyr and Lankinen 2002; Moore and Pannell 2011). On the other hand, it is known that multiple paternity is widespread in plants (Pannell and Labouche 2013), suggesting that pollen competition is a common occurrence under natural conditions. Moreover, recent studies indicate new mechanisms of sexual selection in plants, e.g. competition for optimal placement on the pollinator (Cocucci *et al.* 2014) and early male flowering in dioecious species as a means to compete for access to ovules of high-quality female plants (Forrest 2014). Thus, mechanisms of sexual selection may have been overlooked that can help us explain ecology and evolution of plant traits.

### Sexual conflict

**Figure 2.**
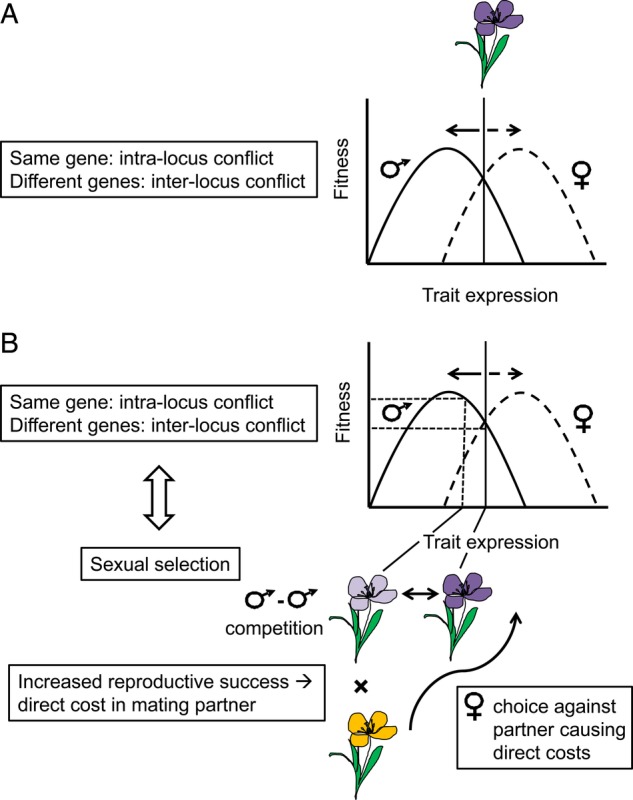
Graphic description of sexual conflict and its relation to sexual selection. (A) Sexual conflict occurs when the male and female evolutionary interests of trait values are opposing, meaning that fitness is maximized for different values in the two sexes or two sexual functions in hermaphroditic species. The conflict can be expressed either between the same gene (intra-locus conflict) or between different genes (inter-locus conflict). (B) Sexual conflict can lead to sexual selection when one mating partner is moving closer to its optimal trait value, which increases reproductive success in competition with other members of the same sex. Because this change in trait value of one mating partner will cause direct fitness costs (e.g. harm) in the other mating partner, this responding mating partner will be selected to discriminate against individuals imposing direct fitness costs, provided that costs connected to such choice is smaller than the costs caused by the harming mating partner. While an ability to mitigate direct fitness costs will be naturally selected, this type of choice mechanism will bias reproductive success of members of the opposite sex, which in essence is similar to mate choice for favourable mates and thus is sexually selected (Arnqvist and Rowe 2005). Moreover, sexual selection for a certain trait value in one sex can generate sexual conflict.

An important development of sexual selection theory has been the increased focus on sexual conflict resulting from opposing trait optima of the two sexes (Parker 1979, 2014; Arnqvist and Rowe 2005; Kokko and Jennions 2014) (Fig. 2A). Sexual conflict can occur either between the same or different genes of males and females (intra- vs. inter-locus conflict, Parker 1979; Rice and Chippindale 2001). Intra-locus conflict concerns when the same trait has different optima in males and females, leading to opposing selection pressure. Inter-locus conflict occurs when the sexes have different optima for an interaction that concerns reproduction (for example when to mate or how often to reproduce). Evolutionary forces will then act on different genes of either sex in order to move trait values closer to their own optimum, which is expected to cause a direct fitness cost in the mating partner. Thus, the critical predictions of inter-locus sexual conflict are selection of sexually antagonistic traits that confer increased reproductive success for the bearer but result in direct fitness costs, such as harm, in the mating partner (Morrow *et al.* 2003), and that these costs can be diminished by a corresponding antagonistic trait in the mating partner (Gavrilets *et al.* 2001; Arnqvist and Rowe 2005). Even though sexual conflict is a broader concept than sexual selection (i.e. sexual conflict does not necessarily involve sexual selection), these two theories are closely connected (Fig. 2B) (Kokko and Jennions 2014). For example, sexual selection for a certain trait that increases mating and fertilization opportunities can generate sexual conflict. Likewise, sexual conflict over trait values can lead to sexual selection if one mating partner has a ‘tool’ that increases its reproductive success while moving its trait value towards its optimum and causing costs in the mating partner (Kokko and Jennions 2014).

Both intra- and inter-locus sexual conflict are expected to occur in hermaphrodite species (Bedhomme *et al.* 2009; Abbott 2011; Schärer *et al.* 2014). In hermaphrodites it is probably relatively more common with an intra-locus conflict where both sexual functions benefit from the same trait values, but there is a negative trade-off between this trait and a second trait in one sexual function (Abbott 2011). In plants, a potential example is when a large inflorescence of several small flowers—rather than fewer large flowers—increase attractiveness to insects and confer higher pollination success for both male and female reproductive functions, but small flowers are negatively correlated with large ovules and healthy seeds. Interestingly, specific inter-locus conflicts may occur in hermaphrodite species that cannot take place in species with separate sexes, including inter-locus sexual conflict over sex allocation, mating roles (acting as female or male during mating) and selfing rates in simultaneous hermaphrodites (Bedhomme *et al.* 2009) and over timing of sex change in sequential hermaphrodites (Schärer *et al.* 2014).

Sexual conflict has been considered a regime shift in animals as it provided a novel way of thinking about male and female mating strategies (Tregenza *et al.* 2006), which has increased our awareness of the conflicting evolutionary interests of the sexes and how this ubiquitous conflict might erode the mutualistic benefits in traditional sexual selection models (e.g. Pischedda and Chippindale 2006). Studies of sexual conflict have substantially contributed to our understanding of evolution of male–female interactions, particularly in insects (Arnqvist and Rowe 2005; Edward *et al.* 2010) but also in higher taxa (fish: Roberts *et al.* 2009; Kwan *et al.* 2013; birds: Royle *et al.* 2002; Tarka *et al.* 2014; mammals: Bro-Jørgensen 2007; Polo *et al.* 2014). Besides explaining maladaptive behaviours and mating decisions (discussed in Pizzari and Snook 2003) the consequences of sexual conflict have been considered from the genetic level to population dynamics and speciation. Inter-locus sexual conflict is believed to result in sexually antagonistic coevolution, or so-called arms-races, between the sexes (Holland and Rice 1998), where the sexually antagonistic traits and behaviours escalate. Such sexually antagonistic coevolution could potentially lead to population differentiation and speciation or to other outcomes, such as cyclic evolution or extensive genetic diversification (Gavrilets and Hayashi 2005). Additional recent topics include, for example, disease dynamics (Gilks *et al.* 2014), ageing (Shi and Murphy 2014), sex determinants (Roberts *et al.* 2009) and the interaction between ecology and sexual conflict (Arbuthnott *et al.* 2014).

In plants, there has been some interest in intra-locus sexual conflict between sexes or sexual functions within the same hermaphrodite or dioecious plant individual  (Fig. 1) (Prasad and Bedhomme 2006). For example, in dioecious *Silene dioica* pollen export and male fitness is benefitted by investment in numerous, small flowers, while seed production and female fitness is benefitted by investment in fewer, but larger flowers (Delph *et al.* 2004). An evaluation of the genetic basis (QTL) of 46 traits in this species revealed that sex-specific QTL contributed relatively more to the genetic variance of traits that were dimorphic, such as flower size, in line with expectations of sexually antagonistic evolution (Delph *et al.* 2010). Another potential sexual conflict at the stage of pollination relates to that high pollen production can be beneficial only for the male function (e.g. increasing pollinator visitation rates, Duffy and Johnson 2011), as indicated by higher female fecundity following emasculation of hermaphroditic plants (Duffy *et al.* 2013; Duffy and Johnson 2014). It has also been suggested that the sexual functions may differ in which pollination system is most favourable; pollination by several different species or pollination by one specialist (Lankinen and Larsson 2009). Even though both sexual functions could benefit from increased pollen transfer in generalist systems, costs of pollen import and export could differ between the sexual functions, e.g. depending of specific pollen and pistil traits (Ashman and Arceo-Gómez 2013). Moreover, during seed provisioning, a sexual conflict may occur because paternal genes should favour higher levels of investment in a seed than maternal genes in cases of multiple paternity (Charnov 1979; Queller 1984; Willi 2013).

Sexual conflict has also been suggested to occur during pollen competition in the pistil  (Fig. 1) (Bernasconi *et al.* 2004), for example over floral receptivity, where selection will favour harmful pollen with an ability to fertilize the ovules ahead of others, securing paternity at the expense of female fitness (Lankinen *et al.* 2006). It is known that pollen deposition can induce floral changes, e.g. wilting of stigma and corolla (O'Neill 1997; Arathi *et al.* 2002; Burkhardt *et al.* 2009). In *Nicotiana attenuata* a post-pollination ethylene burst originating from the stigma and upper style in pollinated flowers was associated with both termination of benzylacetone release which attracts pollinators and augmented differences in siring success between pollen genotypes in mixed pollinations (Bhattacharya and Baldwin 2012). In *Cyananthus delavayi* pollination-induced floral closure was positively connected to the proportion of fertilized ovules, but floral closure caused by natural pollination reduced female fitness in some instances (Niu *et al.* 2011).

#### Sexual conflict over floral receptivity in *Collinsia heterophylla*

It is possible that pistils can delay stigma receptivity in order to reduce a potential fitness cost of pollen-induced shortening of the receptive period (Lankinen *et al.* 2006). In the hermaphrodite annual *C. heterophylla*, plants delay stigma receptivity, which is beneficial for the female reproductive function by enhancing pollen competition for various reasons. Increased competition between self-pollen in *C. heterophylla* (in large pollen loads compared to small ones) reduced inbreeding depression [i.e. impaired offspring fitness due to homozygous deleterious alleles, according to the partial dominance model (Charlesworth and Charlesworth 1987)], suggesting an advantage of avoiding fertilization by low quality self-pollen (Lankinen and Armbruster 2007). Another study on receptive pistils in *C. heterophylla* which compared crosses involving outcross pollen donors, either divided as two pollinations over 2 days or applied as one pollination on the last day, indicated that later fertilization involving simultaneous additions of pollen donors increased offspring quantity (Lankinen and Madjidian 2011). Moreover, following crosses with a mixture of pollen from two donors in both partially receptive and receptive pistils, offspring quality was positively correlated with recipient floral receptivity (Madjidian 2011). The latter trait was estimated in a separate hand-pollination experiment by conducting one-donor crosses in pistils at different developmental stages (Madjidian *et al.* 2012*a*). By cutting off half of the style 4 h after hand-pollination (it takes ∼4 h for pollen tubes to reach the base of the style), only pistils with receptive stigmas and styles at the time of the cross set seeds (see Lankinen and Kiboi 2007), allowing calculation of mean floral receptivity per recipient plant.

Not only recipients, but also pollen donors can influence timing of floral receptivity, i.e. when a pollination can lead to successful seed set (Lankinen and Kiboi 2007). Early fertilization leads to a female fitness cost in terms of reduced seed production and seed biomass (Madjidian *et al.* 2012*b*), which is consistent with a sexual conflict over timing of floral receptivity (Parker 1979; Arnqvist and Rowe 2005). Thus, enhanced pollen competition involving sequential arrival of pollen from several donors appears to be negative for the female reproductive function. The reduction in seed biomass still remained when pollen was applied a second time at a later receptive stage (Madjidian *et al.* 2012*b*). Also, using a small pollen load in the crosses at early stages, compared with a large pollen load, increased seed biomass rather than decreasing it, indicating that early pollinated flowers were not pollen limited. Currently, we do not know the underlying mechanism for the reduced seed set at early fertilization. In fact, it could be caused by other pollen traits apart from an ability to induce stigma receptivity. For example, if not only the stigma but also the ovule receptivity is delayed, fast growing pollen tubes in unripe pistils could cause the low seed set by arriving early at the ovary. Provided that early arrival to the ovary is beneficial in terms of increased siring success despite the lowered number of sired seeds, rapid growth of pollen tubes would be the sexually antagonistic trait, as it is increasing competitive ability and causing pistil harm.

While most studies on the potential sexual conflict over timing of floral receptivity in *C. heterophylla* have been conducted in the greenhouse, a recent field study confirmed that flowers at early stages produce nectar and receive pollinator visits which can lead to seed set (E. Hersh, J. A. Madjidian, S. Andersson, M. Strandh, W. S. Armbruster, Å. Lankinen, unpubl. data). Thus, there is opportunity for the conflict to be of significance also under natural conditions. A piece of evidence for a history of antagonistic evolution between pollen and pistils within populations of *C. heterophylla* comes from a study involving four populations originating from two regions in California (Madjidian and Lankinen 2009). Experimental crosses between and within populations and regions showed that pollen source affected timing of floral receptivity. Pollen induced earlier receptivity on another population than their own within the same region, but not between regions where receptivity instead was later. Moreover, the cost of early fertilization was only present following crosses on plants from the same region.

## Incorporation of sexual selection and sexual conflict in plant studies

In order to better incorporate the theories of sexual selection and sexual conflict in plant studies, an important first step is to learn about the complexity of these theories and how to measure them in our own study systems. Jones and Ratterman (2009) point out that the main progress of the research field of sexual selection is based on three approaches: theoretical modelling of mate choice mechanisms, molecular techniques to study parentage in natural populations and measurement of selection differentials through quantitative genetics. In an extensive review on phenotypic selection in natural populations across taxa (plants included), Kingsolver *et al.* (2001) found that sexual selection was often stronger than natural selection. Although there might be a bias in these numbers (for example that sexual selection studies have been made on species having some feature that indicates a high potential for sexual selection) these results imply that sexual selection might have a vast evolutionary effect. In many cases, natural and sexual selection interact; Scordato *et al.* (2014) found, for example, that it was most often a combination of natural and sexual selection involved in speciation. Similarly, ecology and sexual selection can jointly influence the evolutionary response (e.g. Karlsson *et al.* 2010; Arbuthnott *et al.* 2014; Green *et al.* 2014). Some plant studies have aimed to measure the relative magnitude of sexual selection vs. natural selection, i.e. fecundity or viability selection for trait values (Emms *et al.* 1997; Delph and Herlihy 2012), which indeed can be an important approach. However, it could also be valuable to test different hypotheses regarding which selective forces are at play. For example in animal biology, features of the immune system that traditionally have been considered as adaptations to fecundity selection are in fact now discussed to instead be sexual conflict adaptations (Morrow and Innocenti 2012).

Sexual conflicts are notoriously difficult to study as the evolutionary forces of males and females often balance each other under natural conditions, and are difficult to separate from other evolutionary forces on male–female interactions (Rowe and Day 2006). Some studies on both animals and plants have used crosses between and within populations of the same species (e.g. Andrés and Arnqvist 2001; Jolivet and Bernasconi 2007), where the reasoning is that antagonistic evolution is easier to detect when male–female adaptations are mismatched due to differences in the outcome of inter-sexual coevolution in different populations (Parker and Partridge 1998; Andrés and Arnqvist 2001). However, this methodology has been criticized, e.g. because the outcome can also depend on population history (Rowe *et al.* 2003; Pizzari and Snook 2004; Rowe and Day 2006). An approach that has proved more successful for studying sexual conflict, particularly in insects but not yet performed in plants, comes from experimental evolution studies varying the degree of sexual selection artificially, e.g. enforced monogamy vs. polyandry (Rice 1996; Edward *et al.* 2010). Additionally, other types of studies conducted under more natural conditions are important, such as determining the key elements of sexual conflict—reproductive success of male and female conflict traits and conflict costs connected to these—either by direct estimation or through manipulative studies (e.g. Arnqvist and Rowe 1995; Watson *et al.* 1998, see also Arnqvist and Rowe 2005; Rowe and Day 2006).

In plants, we argue that we need to consider the possibility of sexual conflict despite its complexity but take care to design accurate experiments where existence of antagonistic evolution can be revealed. For example, while multiple mechanisms can select for pistil traits that enhance pollen competition, as outlined above, most studies assume that enhanced pollen competition is always positive for the reproductive function (see Pannell and Labouche 2013). It would be important to investigate co-occurrence of both positive and negative effects of enhanced pollen competition, for example the joint effects of sexual selection and sexual conflict, i.e. indirect and direct selection on mate choice in plants.

## Examples of Research Fields that Could Benefit from Considering Theories of Sexual Selection and Sexual Conflict

### Mechanisms of pollen–pistil interactions

Because the opportunity for sexual selection and sexual conflict is considered to be particularly large during pollen competition, it would seem evident to take these theories into account when studying interactions, communication and other physiological mechanisms of pollen germination and growth in the pistil. From studies on animals it is known that sperm competition often gives rise to sexual conflict; the male ejaculate is adapted to male–male competition within the female reproductive tract and these adaptations might have a harmful side effect on females leading to sexual conflict and consequently female antagonistic adaptations (e.g. Schärer *et al.* 2011, see review by Stockley 1997).

Even though some studies have considered mechanisms of pollen and pistil interactive traits in relation to pollen competitive ability and the potential positive effects of enhancing pollen competition (Hormaza and Herrero 1992; Erbar 2003; Sanchez *et al.* 2004), most studies on pollen–pistil interactions have focussed on incompatibility reactions between or within species, or pollen tube tip-growth and guidance in the pistil as a means of efficient delivery of sperm cells to the ovules (see special issues Hiscock 2011; Cheung *et al.* 2013). It is, however, conceivable that pollen traits have evolved in order to increase pollen competitive ability at the expense of harming the pistil tissue or causing other direct costs. Pistil ability to prevent potentially suboptimal pollen tube routes or mitigate harmful pollen growth could also be expected.

Main innovations of the angiosperms concern reproductive biology during the phase between pollination and fertilization, such as small ovules, the closed carpel and rapid pollen tube growth made possible by callosic walls (Williams 2008). Interestingly, it appears that rapid pollen tube growth rate preceded the closed carpel and could have influenced diversification of carpel form and function (Williams 2008). For example, fusion of floral carpels (syncarpy), another key innovation in angiosperms (Endress 2001), often allows for pollen tubes to cross between ovules, increasing seed set, and is associated with enhanced levels of pollen competition, possibly increasing offspring fitness (Armbruster *et al*. 2002*a*). Moreover, pollen germination rate and tube growth rate has been shown to be faster in evolutionary derived flowering seed plants compared with in early divergent angiosperms (Williams 2012*a*, *b*). It could be hypothesized that selection of pollen tube paths that increase reproductive success in the case of pollen competition could be harming the pistil tissue as a side effect, in line with a sexual conflict. In fact, in order to invade pistil tissue pollen tubes need to soften the tissue using enzymes and proteins, which can sometimes be brought about by triggering an autodigestion mechanism (reviewed by Sanati Nezhad and Geitmann 2013). It is known that the pistil can control pollen tube growth in the stigma, the canal or transmitting tract of the style or the ovary by both providing and restricting support, the latter, e.g. brought about by reducing the resources available to growing pollen at the base of the style, enhancing pollen competition (Herrero and Hormaza 1996; Herrero 2003; Losada and Herrero 2014). Interestingly, a comparison of pollen tube pathways in the pistil in ancient angiosperm species with one to three ovules has shown a difference in pollen tube growth in their open carpels, sealed with secretion rather than tissue; In *Amborella* (Amorellaceae) only a few pollen tubes enter the open mouth of the stylar canal (Williams 2009), while in *Brasenia* and *Cobomba* (Cabombaceae) pollen tubes do not enter through the open mouth but instead reach the canal by growing directly through the carpel wall allowing more pollen tubes to enter (Taylor and Williams 2009). It would be interesting to consider if this growth pattern induce costs for the female reproductive function. Such a scenario would be parallel to traumatic insemination in bed bugs, where male insemination occurs through the body wall rather than through the reproductive tract (Stutt and Siva-Jothy 2001). In bed bugs, females have evolved a special structure in the body wall (spermalege) that reduces the risk of mating-associated infection and thus increases female tolerance to male mating behaviour (Reinhardt *et al.* 2003).

In *Dalechampia* ssp. the stigmatic surface is extended down the sides of the style so that some pollen arrives much closer to the ovary than others (Armbruster *et al.* 1995). However, the pistil is controlling pollen tube growth so that all pollen has to grow to the tip of the style before starting to grow towards the ovules, suggesting a cost for the growing pollen tubes. Similarly, self-incompatibility systems are often controlled by the pistil rather than pollen, as pollen capacity to self-pollinate is dependent on the pistil environment (reviewed in Brandvain and Haig 2005). In fact, theoretical arguments indicate that the high diversity commonly found for self-incompatibility alleles should result from selection for uncommon alleles in pollen as it will increase pistil acceptance (De Jong and Klinkhamer 2005) suggesting a conflict over selfing rate. Curiously, in the domesticated apple, where the style controls pollen tube growth by reducing both growth area and supporting β-glucans and extensins along the style, pollen tubes arrive in two waves (Losada and Herrero 2014). It is not known which pollen fertilize the ovules or if this opportunity of pollen selection first in the style and second at the ovary site represents a fine-tuned selection mechanism that is beneficial for the female reproductive function. In *C. heterophylla*, hand-pollination on fully receptive stigmas performed on two consecutive days (24 h apart) resulted in some seeds sired from the second pollen load, despite that it takes only between 2 and 4 h for pollen to grow down the style (Lankinen and Madjidian 2011). Potentially, the pistil has a selection mechanism that favours diversity of the fertilized seeds (Bernasconi *et al.* 2003). Enhancing pollen competition by hand-pollinating fully receptive stigmas only once at a late stage, as opposed to earlier and twice over 2 days, increased offspring quantity and genetic diversity in *C. heterophylla* (Lankinen and Madjidian 2011).

Interestingly, in *Brassicaceae* rapid evolution of pollen coat proteins involved in pollen germination was found (Fiebig *et al.* 2004), potentially suggesting antagonistic interactions with the pistil concerning germination. A recent study comparing gene expression in sporophytes, pollen and sperm in *Capsella grandiflora* found support for strong purifying selection in genes only expressed in pollen (Arunkumar *et al.* 2013). It would be appealing to perform more direct tests of fitness costs and benefits in relation to cellular mechanisms of pollen–pistil interactions as well as differential gene expression in pollen and pistil (both in the absence and presence of one another) in order to test hypothesis of sexual selection and sexual conflict.

### Evolution of mating-system traits and mixed-mating in hermaphrodites

In animals, mating systems concern whether individuals are monogamous, polygamous or promiscuous, and studies might focus on the evolutionary effects of certain mating systems (e.g. Rowe *et al.* 1994; Smith and Härdling 2000; Härdling and Karlsson 2009). However, interestingly, Jones and Ratterman (2009) point out that the relation between mate choice and mating-system evolution is in general understudied. In hermaphroditic species, mating systems also involve fertilization by another individual vs. self-fertilization (outcross- and self-pollination in plants). While theory predicts that selection should favour hermaphrodite individuals with either complete outcrossing or complete self-fertilization (Lande and Schemske 1985), mixed-mating, i.e. a combination of outcross pollination and self-pollination in the same individual, is often found in insect-pollinated plant species (Goodwillie *et al.* 2005). Substantial research has proposed that mixed-mating is either a transitional stage (Lande and Schemske 1985) or evolutionary stable (Lloyd 1979; Holsinger 1991; Porcher and Lande 2005; Harder *et al.* 2008; Johnston *et al.* 2009; Winn *et al.* 2011; Devaux *et al.* 2014). Transitions from outcrossing to selfing are commonly connected with a suite of floral traits, such as reduced flower size, loss of spatial and temporal separation of male and female structures and increased developmental rate (Runions and Geber 2000; Totland and Schulte-herbrüggen 2003; Herlihy and Eckert 2007; Goodwillie *et al.* 2010). However, some of these traits are influenced by additional selective forces, which are important to consider in order to gain knowledge about evolution of mating-system and correlated traits. One example is that stressful conditions can favour rapid floral development (time-limitation hypothesis, Aarssen 2000). Moreover, some floral traits associated with mating system could be affected by sexual selection/sexual conflict, e.g. flower size and timing of stigma receptivity, but these selective forces are seldom taken into account in studies of mating-system evolution. Interestingly, it has been predicted that due to stronger sexual selection between pollen donors in outcrossing plants, pollen should be better at competing in more outcrossing taxa, e.g. germinate and grow faster pollen tubes and perform more consistently across pistil environments (Mazer *et al.* 2010). Investigating selfing and outcrossing species of *Clarkia* showed only partial support for these predictions, suggesting that additional factors influence pollen competitive ability in these species (Hove and Mazer 2013). In *Arabidopsis lyrata*, seeds were larger following between-population crosses when the pollen source was from another outcrossing population compared to from a selfing population or from the same outcrossing population as the recipient (Willi 2013). This result can be interpreted as high pollen influence on seed provisioning in outcrosses only when recipients had not co-evolved with pollen, which is in line with male–female sexual antagonism regarding seed provisioning.

In order to discuss how consideration of sexual conflict may influence our understanding of evolution of mating-system and correlated traits, we will use the example of mixed-mating *C. heterophylla* and the sexual conflict over timing of stigma (floral) receptivity (as previously outlined), a floral trait that is linked to the mating system.

#### Pollen competition, sexual antagonism and mating-system evolution in *Collinsia heterophylla*

In the genus *Collinsia* (ca. 22 species) variation in mating system and outcrossing rate is large, constituting one group of mainly selfing species and one group of more outcrossing, mixed-mating species (Armbruster *et al*. 2002*b*; Kalisz *et al.* 2012). The more outcrossing group has typically larger flowers, delayed selfing and delayed stigma receptivity. Transitions usually from large to small flowered species are also known to have occurred (Armbruster *et al*. 2002*b*; Baldwin *et al.* 2011). In large-flowered *C. heterophylla* populations vary considerably in outcrossing rate, ranging between 0.32 and 0.64 based on allozyme markers and microsatellite markers (Charlesworth and Mayer 1995; Kalisz *et al.* 2012) and between 0.62 and 0.94 based on morphological markers (Weil and Allard 1964). Populations also show high variability in mating system-related floral traits, such as stage of stigma receptivity (Armbruster *et al*. 2002*b*; Madjidian and Lankinen 2009; Å. Lankinen, J. A. Madjidian, S. Andersson, unpubl. data). At the inter-specific level of *Collinsia*, stage of stigma receptivity, i.e. timing of stigma receptivity in relation to days (= number of open anthers) after flower opening, was recently shown to be the only trait that was significantly correlated with outcrossing rate (Kalisz *et al.* 2012), suggesting that understanding how selection acts on this trait can be of importance for learning about mating-system evolution in *Collinsia*.

Delayed stigma receptivity may be advantageous in more outcrossing species due to separation of male and female function in time (dichogamy), preventing self-pollination in the presence of pollinators (Darwin 1877; Lloyd and Webb 1986). However, because self-pollen arriving to unreceptive stigmas can fertilize the ovules upon receptivity in *C. heterophylla* (Lankinen *et al.* 2007), it is more likely that the adaptive reason for possessing this trait for the female reproductive function is to enhance pollen competition either between self or between outcross pollen (Lankinen and Armbruster 2007; Lankinen and Madjidian 2011). Enhancing pollen competition between self-pollen can be of particular importance in a mixed-mating species as inbreeding depression can be reduced by avoiding fertilization by low quality self-pollen, lowering the cost of selfing in a mixed-mating species and potentially favouring stability of mixed-mating (Armbruster and Rogers 2004).

Because we identified a sexual conflict over timing of floral receptivity in *C. heterophylla* (Lankinen and Kiboi 2007; Madjidian *et al.* 2012*b*), which could lead to sexually antagonistic evolution (Madjidian and Lankinen 2009) it would be of interest to consider a potential interrelation between mating-system evolution and antagonistic evolution on timing of stigma receptivity. Selection on the ability of pollen to induce early receptivity, causing the direct cost of reduced seed production (Madjidian *et al.* 2012*b*) should be strongest in more outcrossing populations. Given that delaying stigma receptivity can reduce the cost of early seed production, we should expect that highly outcrossing populations express more pronounced conflict traits, and increased sexually antagonistic evolution of pollen and pistil. Because one possible outcome of antagonistic coevolution is temporal cycles of exaggeration and reduction of conflict traits (Gavrilets and Hayashi 2005; Rowe *et al.* 2005), it is conceivable that antagonistic selection could lead to acceptance of early arriving pollen, i.e. early stigma receptivity in some populations in order to escape conflict costs. However, because early stigma receptivity does not enhance pollen competition, this outcome could be hypothesized to reduce possibilities to lower inbreeding depression in the case of self-pollination, decreasing stability of mixed-mating. Preliminary results of a study investigating conflict costs and male vs. female influence on stage of floral receptivity following hand-pollinations across 12 populations of *C. heterophylla* that differed in outcrossing rate, found large variation in both conflict costs and male vs. female influence across populations but variation was independent of outcrossing rate (E. Hersh, J. A. Madjidian, S. Andersson, M. Strandh, W. S. Armbruster, Å. Lankinen, unpubl. data). We also found that pollen tube growth rate was uncorrelated with outcrossing rate, contrary to expectation (Mazer *et al.* 2010). Curiously populations with a significant male influence on floral receptivity had faster pollen tube growth rates and earlier stigma receptivity, as indicated by tests of peroxidase activity in the absence of pollen. We can hypothesize that this unexpected link between traits—we would rather anticipate that coordinated germination by many pollen grains due to late stigma receptivity would select for fast pollen tube growth rate—is in some way related to sexually antagonistic selection. Moreover, despite the earlier stigma receptivity in these populations, floral receptivity, i.e. the stage when pollination resulted in seed set did not differ among populations. In future studies it would be valuable to investigate if the detected discrepancy between timing of stigma receptivity and timing of first seed set represents a female resistance mechanism in populations where pollen impose high costs by early fertilization, as would be expected from a sexual conflict scenario (Parker 1979; Arnqvist and Rowe 2005).

Even though we clearly need additional studies to understand the potential interrelation between pollen competition, sexual conflict and mating-system evolution in mixed-mating *C. heterophylla*, this exemplifies how taking evolutionary forces generated by sexual selection/sexual conflict into account can introduce an additional dimension to studies on mating-system evolution in hermaphrodites.

### Plant immune responses to pests and pathogens

In animals, an association between sexual selection and disease defence was suggested already in the 1980s (Hamilton and Zuk 1982). A large number of animal studies have investigated mate choice in relation to direct (e.g. avoidance of infected mates) and indirect (e.g. mates carrying genes conferring increased disease defence in offspring) benefits (Andersson 1994; Beltran-Bech and Richard 2014). In some animals, MHC, the major-histocompatibility complex (MHC) responsible for self-recognition in the adaptive immune system (Klein 1986), is involved in mate choice (Penn and Potts 1999; Olsson *et al.* 2003; Ziegler *et al.* 2005). Males are believed to be chosen by females either because (i) they have high or optimal MHC diversity, which increase recognition of pathogens in offspring (Potts *et al.* 1991; Grob *et al.* 1998), (ii) they have complementary MHC genes (Tregenza and Wedell 2000; Strandh *et al.* 2012) or (iii) they have specific MHC haplotypes with, for example, beneficial effects in particular environments (Von Schantz *et al.* 1996). Recent studies also suggest that intra-locus sexual conflict can have important effects on sex differences in disease genetics (see review by Gilks *et al.* 2014) and that intense sexual selection and sexual conflict might reduce investment in the immune function (van Lieshout *et al.* 2014).

Plant defence to pests and pathogens includes both constitutive (passive) barriers and inducible responses. While innate plant immune responses, causing inducible responses, are non-adaptive, recent molecular tools have revealed multiple recognition mechanisms and signalling pathways involved in pathogen infectivity and plant defence interactions and coevolution (Dangl *et al.* 2013). In plants, however, a link between defence responses and sexual selection is rarely considered.

The fact that plants are sessile and mate by proxy constitute increased risks of attracting antagonistic organisms. Floral traits that increase pollinator visits can also attract antagonistic insects and vectors for pathogens (Strauss and Whittall 2006; McArt *et al.* 2014), and infection risk can differ between male and female plants (Shykoff *et al.* 1996; Kaltz and Shykoff 2001). Thus, selection for, for example, large flowers can be affected by a compromise between sexual selection to attract pollinators and natural selection to avoid pathogens (Galen and Cuba 2001; Elzinga *et al.* 2007; Burkhardt *et al.* 2012), in either sexual function (see Box 1). Several traits connected with reproductive success are also indirectly negatively affected by herbivory and diseases, including flower morphology and pollinator visits (Strauss *et al.* 1996), and pollen competitive ability (Stephenson *et al.* 2003). However, because pollen performance is affected by health status of the paternal sporophyte plant, enhanced pollen competition could favour mate choice for fertilization of pollen produced by the most resistant or tolerant plants (Andersson 1994). On the other hand, disease resistance could also confer costs (Strauss *et al.* 2002), potentially affecting pollen competitive ability. In healthy *Cucumis sativus*, siring success following two-donor crosses did not differ between pollen produced by plants that were either resistant or susceptible to yellow mosaic virus, but in infected plants, susceptible pollen was more successful (Kiboi and Skogsmyr 2006). This result may have been caused by cost of resistance in pollen related to post-fertilization effects, as pollen tube growth rate did not differ between pollen types. Curiously, pistils of infected plants showed increased nutrient levels in the pistil, which could have influenced provisioning of developing seeds. Another study on *C. sativus* showed that soil nutrients were positively correlated with nutrients in the pistil, which influenced relative pollen competitive ability of pollen donors grown under similar nutrient conditions (Haileselassie *et al.* 2005).

Insect vectors can transfer pathogens directly to flowers, e.g. smut-fungus in *Silene*, but also transmission of pathogens by wind dispersal of spores can occur during the flowering phase (Antonovics 2005). Pathogens often enter plants through the nectaries of flowers (Sasu *et al*. 2010*a*) or through the pistil (Ngugi and Scherm 2006), the latter by producing infectious units that circumvent plant immune responses by mimicking pollen tubes invading the pistil (Ngugi and Scherm 2004; Kessler *et al.* 2010). In *N. attenuata* plants with defective ethylene signalling allowed growth of colonizing root microbes (Long *et al.* 2010), and also appeared to have pistils that were less discriminating between competing pollen donors (Bhattacharya and Baldwin 2012), suggesting a similar role for ethylene signalling in the case of growing pollen tubes and growing hyphae. Plants can also defend themselves against antagonists by producing deterring floral volatiles reducing flower feeding insects (Junker and Blüthgen 2008; Kessler *et al.* 2008; Willmer *et al.* 2009), volatile organic compounds functioning as a fumigant that limits bacterial growth on the stigma (Huang *et al.* 2012), and anti-microbial chemicals and proteins in nectar (Hillwig *et al.* 2010; Sasu *et al*. 2010*b*). Also flavonoids, that accumulate on wounded and damaged stigmas and have anti-microbial effects, have been speculated to have a function in pathogen resistance (Stephenson 2012). Interestingly, flavonoids influence pollen germination and tube growth (Taylor and Hepler 1997).

It could be hypothesized that effectiveness of plant defence is influenced by differences in pistil environment, either due to compatibility reactions or pistil traits with the capacity to favour some pollen over others or mitigate conflict costs caused by antagonistic pollen. Interestingly, a recent study on the Solanaceae family found that species with self-incompatibility systems had relatively more constitutive defence to herbivores while self-compatible species had relatively more induced defence (Campbell and Kessler 2013). It was assumed that this difference was connected to mating system (outcrossing vs. selfing) because selective forces favouring selfing (e.g. marginal habitats or range edges with a lack of pollinators) would also favour phenotypic plasticity. Moreover, induced defence may be costly in taxa dependent on pollinating insects, as induced defence responses can lead to changes in plant scents attracting pollinators (Kessler *et al.* 2011). However, neither pollination dependence nor outcrossing rates of self-compatible species were estimated in this study. It should be interesting to know whether the difference in defence between incompatible and compatible species is influenced by reactions in self-incompatible vs. self-compatible pistils, or competitive pollen and pistil traits connected to the difference in pistil environment.

In many animal taxa post-mating activation of the immune system can occur independent of damage or pathogen presence following mating. Hypotheses explaining this activation include preparation for embryo implantation and responses to increased probability of pathogen infection or physical harm, but also the more recent idea that male reproductive proteins can activate the female immune system, as suggested in mammals and fruit flies (reviewed by Morrow and Innocenti 2012). Because male reproductive proteins potentially are involved in male–male competition at the post-mating stage, it is possible that not only natural selection but also sexual selection/sexual conflict may be directly involved in immune system evolution. So far, this hypothesis needs to be empirically tested in more detail but it would be very interesting to consider also in plants. For example, a start would be to investigate pistil immune responses after hand-pollination in study systems where pollen competition is common and compared with systems where it is rare, as this effect should only be found when sexual antagonism is present (Morrow and Innocenti 2012). Detailed investigation of immune genes would also be important, such as testing the prediction of higher rates of evolution for some immune genes caused by antagonistic evolution between pollen reproductive traits and pistil immune responses.

## Conclusions

Here, we have discussed that even though some researchers are applying the theories of sexual selection and sexual conflict to plant studies today, these theories are not considered as important and useful as in animals where these theories were first developed (Darwin 1871; Parker 1979). We think that there may be a combination of reasons for the relatively few such studies in plants. Even though sexual selection traditionally has been controversial in plants, accumulating evidence for sexual selection and sexual conflict in plants seems to have increased acceptance for these theories. However, one difference between animals and plants is that the majority of intra-sexual competition and mate choice in animals takes place before mating, which means that researchers studying animal behaviour are constantly intrigued (as Darwin was) and reminded of these processes. Interestingly, the use of genetic markers provided a major breakthrough in the study of sexual selection, allowing for tests of paternity in natural populations but also increased opportunities for studies of post-copulatory processes during both sperm and pollen competition. However, advances of the more accurate measures of biological process would never have been successful without a theoretical framework directing which hypotheses to test. This theoretical framework is complex but essential in order to develop plausible mechanisms for plants and to design accurate experiments in plant biology. To follow the advancements in sexual selection and sexual conflict, an interdisciplinary interest in animals, where the theory is constantly developing, might be needed, which could be another possible reason for the low interest in sexual selection and sexual conflict in plant biology. In fact, it has been proposed that when sexual selection was first suggested in plants, it was the use of animal-oriented words and direct comparisons across taxa in combination with efforts to publish in more general scientific journals that influenced many plant researchers to start thinking of plant reproduction in new ways, which in turn generated a wealth of studies (Thomson 2014).

We believe that much can be gained from fully acknowledging that sexual selection and conflicting male and female interests are important beyond species where we can easily observe mating behaviour. Considering sexual selection and sexual conflict theories also in a wider range of plant studies on ecology and evolution can be useful, for example in the three research areas we have discussed here. Increased knowledge about mechanisms of pollen–pistil interactions in relation to male–male competition and female choice is of particular interest as we expect direct interactions between competing pollen and pistil tissue. By studying sexual selection on haploid pollen, we have also the opportunities to learn about differences in adaptation in relation to haploid vs. diploid selection (cf. masking hypothesis, Kondrashov and Crow 1991). Mating-system evolution in hermaphrodites is after considerable research still incompletely understood (e.g. Goodwillie *et al.* 2005; Winn *et al*. 2011; Devaux *et al.* 2014). Even though we do not know if sexual selection can influence mating-system evolution, several mating-system related traits (e.g. pistil traits or floral traits influencing pollination success) are also targets for sexual selection and conflict. Interestingly, in animals the interrelation between the evolution of mate choice and mating-system evolution is understudied (Jones and Ratterman 2009). Finally, plant immune responses to pests and pathogens is an important field, from which insights often can be directly used in breeding programmes to improve plant protection in crops. Studies of insect pests, e.g. host plant choice, commonly take into consideration that male and female behaviour also is influenced by sexual selection. In fact, sex pheromones that many insects use for mate location is successfully exploited for monitoring and mass-trapping (Witzgall *et al.* 2010). On the other hand, in studies of resistance and tolerance to pests and pathogens, plants are often considered as completely unaffected by any differential selective forces on male and female functions. We argue that applying these theories more broadly in plants will challenge our understanding of plant ecology and evolution, and could contribute to important general insights about adaptation related to differential selective forces acting on males and females.

## Sources of Funding

The Swedish Research Council supported this work.

## Contributions by the Authors

Å.L. wrote the first draft. The text was improved by both authors.

## Conflicts of Interest Statement

None declared.
